# Visibility evaluation of colorectal lesion using texture and color enhancement imaging with video

**DOI:** 10.1002/deo2.90

**Published:** 2022-01-11

**Authors:** Naoto Tamai, Hideka Horiuchi, Hiroaki Matsui, Hiroto Furuhashi, Shunsuke Kamba, Akira Dobashi, Kazuki Sumiyama

**Affiliations:** ^1^ Department of Endoscopy The Jikei University School of Medicine Tokyo Japan

**Keywords:** colorectal cancer, image‐enhanced endoscopy, narrow‐band imaging, texture‐enhanced imaging

## Abstract

**Objective:**

To evaluate the visibility of colorectal lesions using a novel image processing algorithm, texture and color enhancement imaging (TXI), that allows the acquisition of brighter images with enhanced color and surface structure.

**Methods:**

During August–September 2019, patients referred for endoscopic treatment were prospectively recruited. Electronic data acquired while observing colorectal lesions using white light imaging (WLI) were obtained and recorded: WLI, TXI mode1 (with color enhancement), and TXI mode2 (without color enhancement) videos were constructed. The lesions were also recorded using narrow‐band imaging (NBI) from the same perspective as WLI. Four video clips (WLI, TXI mode1, TXI mode2, and NBI) were made per lesion. Thereafter, video files for evaluations were prepared by randomly arranging all video clips. Finally, visualization scores were evaluated by four endoscopists, and the WLI, TXI mode1, TXI mode2, and NBI results were compared.

**Results:**

Overall, 22 patients with 68 lesions were recruited; the video file for evaluation subsequently comprised 272 randomly arranged video clips. Mean visualization scores using WLI, TXI mode1, TXI mode2, and NBI were 70.0 (±20.1), 80.5 (±18.6), 75.6 (±18.1), and 69.0 (±20.6), respectively. Mean visualization scores for flat lesions using WLI, TXI mode1, TXI mode2, and NBI were 64.1 (±21.2), 76.5 (±20.18), 71.8 (±19.4), and 64.2 (±22.0), respectively. Visualization scores using TXI mode1 were significantly better than those using WLI, TXI mode2, or NBI.

**Conclusions:**

TXI enables improved visualization of colorectal lesions, even flat lesions, than WLI and NBI. TXI may allow better detection of colorectal lesions, although further prospective studies are required.

## INTRODUCTION

The incidence of post‐colonoscopy colorectal cancer is estimated to be 0.7–1.7 cases per person‐years,^1^, ^2^ and 40%–50% of these cases are believed to be caused by missed lesions.^3^ These missed lesions can be categorized as “lesions in non‐visual field” or “lesions in visual field.” To reduce the incidence of missed lesions in the non‐visual field, wide‐view scope and distal attachments have been applied to colonoscopy with positive results.^4^, ^5^, ^6^, ^7^ To reduce the incidence of missed lesions in the visual field, the efficacy of image‐enhanced endoscopy (IEE) has been improved using novel techniques, such as narrow‐band imaging (NBI), autofluorescence imaging, linked color imaging, and so forth.^8^, ^9^, ^10^, ^11^, ^12^, ^13^ These have been evaluated in many studies; however, reports on their efficacy in detecting colorectal lesions have been conflicting. Recently, a novel IEE technique, texture and color enhancement imaging (TXI), was developed by the Olympus Corporation (Tokyo, Japan). This study aimed to determine whether TXI can improve the visibility of colorectal lesions.

## METHODS

**FIGURE 1:**
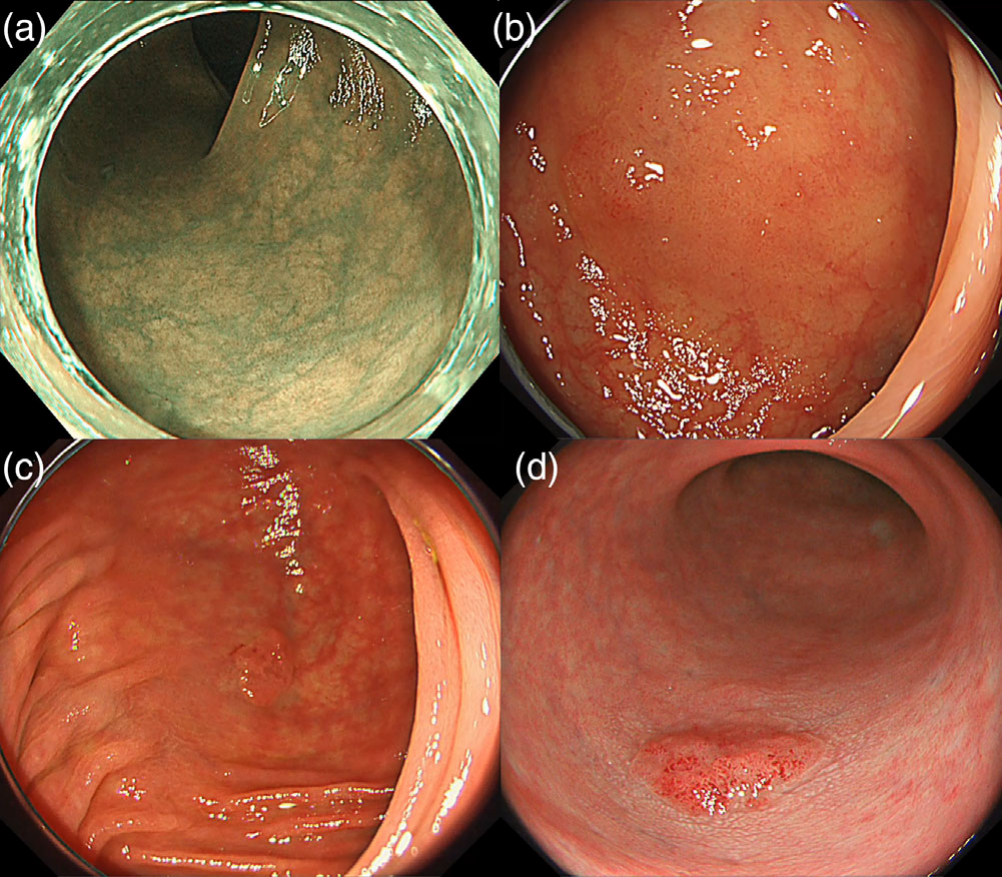
Examples of the evaluated image. (a) NBI image of hyperplastic polyp scored as 0–25 based on a visual analog scale. (b) WLI image of Intramucosal adenocarcinoma scored as 25–50 based on a visual analog scale. (c) TXI mode2 image of tubular adenoma scored as 50–75 based on a visual analog scale. (d) TXI mode1 image of tubular adenoma scored as 75–100 based on a visual analog scale. NBI: narrow‐band imaging; WLI: White light imaging; TXI: texture and color enhancement imaging

This study was a retrospective study using prospectively collected videos. The study protocol was approved by the research ethics committee of our institution and was registered in the University Hospital Medical Network Clinical Trials Registry (UMIN 000039073). From August to September 2019, patients were prospectively recruited. The inclusion criteria of this study were as follows: the patients with known colorectal lesions, the patient who was directly referred to the Endoscopy division of the Jikei Medical University Hospital, and the patient agreed to receive endoscopic treatment and to be enrolled in this study. Written informed consent for endoscopic treatment and enrollment of this study was obtained from all patients before enrollment in this study. All patients were administered a polyethylene glycol solution and underwent colonoscopy using a video processor system (EVIS LUCERA ELITE system; Olympus Corporation) and a colonoscope (PCF‐H290; Olympus Corporation). The electronic data obtained during observation of colorectal lesions using white light imaging (WLI) were recorded. WLI, TXI mode1 (with color enhancement), and TXI mode2 (without color enhancement) videos were constructed using the recorded electronic data. In addition, the lesions were observed and recorded using narrow‐band imaging (NBI) from the same view as WLI, and NBI videos. Four video clips (WLI, TXI mode1, TXI mode2, and NBI) were made per lesion. Thereafter, video files for the evaluation were made by randomly arranging all video clips. Finally, visualization scores were evaluated by four endoscopists (two experts with the experience of IEE >1000 and two trainees with the experience of endoscopy <1000) on the basis of a visual analog scale (0, recognition of the lesion was judged as worst; 25, recognition of the lesion was judged as poor; 50, recognition of the lesion was judged as acceptable; 75, recognition of the lesion was judged as good; and 100, recognition of lesion was the best.) (Figure 1). The evaluations were conducted using the personal computer and the distance from the monitor to the rater was about 70 cm. These scores were compared among the WLI, TXI mode1, TXI mode2, and NBI. The primary outcome of this study was the visualization scores of colorectal lesions for each modality, and the secondary outcome was the visualization scores of flat colorectal lesions using each modality.

### Principles of TXI

TXI is a novel IEE technique developed by Olympus Corporation. TXI was developed to improve lesion visibility by enhancing three image factors of WLI: (1) brightness in the dark area, (2) texture such as subtle surface elevation or depression, and (3) color such as slight color changes in the image. TXI was designed to enhance these factors by applying an image processing technology based on the Retinex theory.^14^ The main principle of this theory is the decomposition of images into two layers, according to the characteristics of the human vision system^15^: the first is a base layer that represents the illumination light in the scene; the other is a detailed layer corresponding to the local contrast of brightness and color in the scene.

**FIGURE 2:**
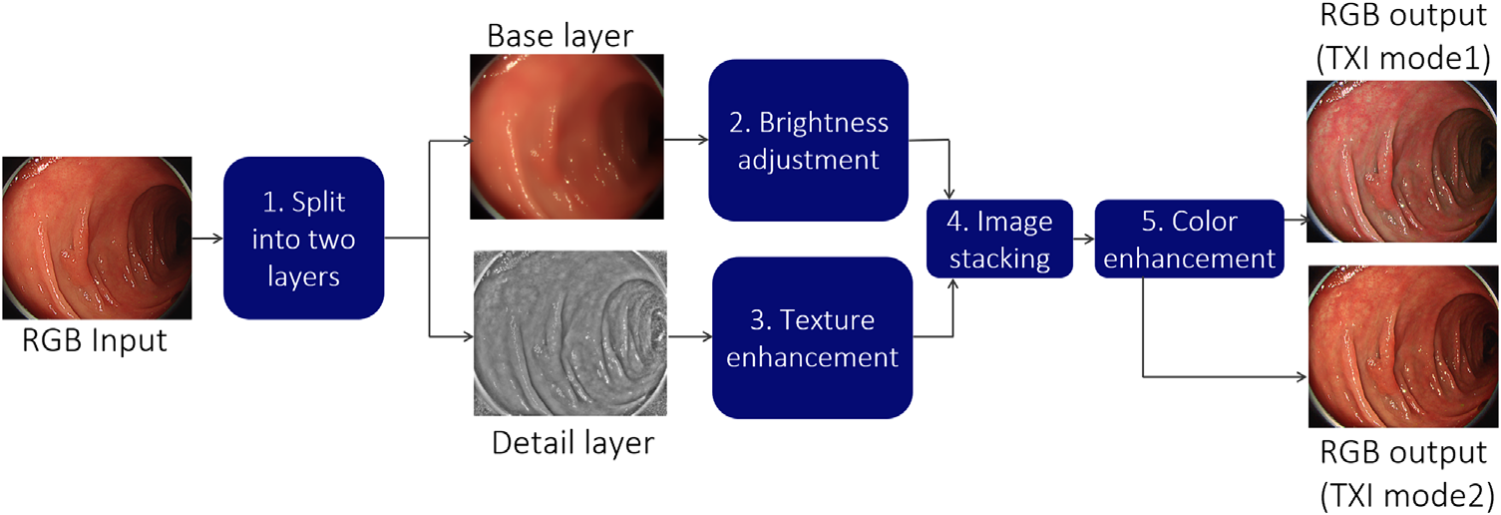
Principles of texture and color enhancement imaging. TXI: texture and color enhancement imaging

The flowchart of TXI principles is shown in Figure 2.

**FIGURE 3:**
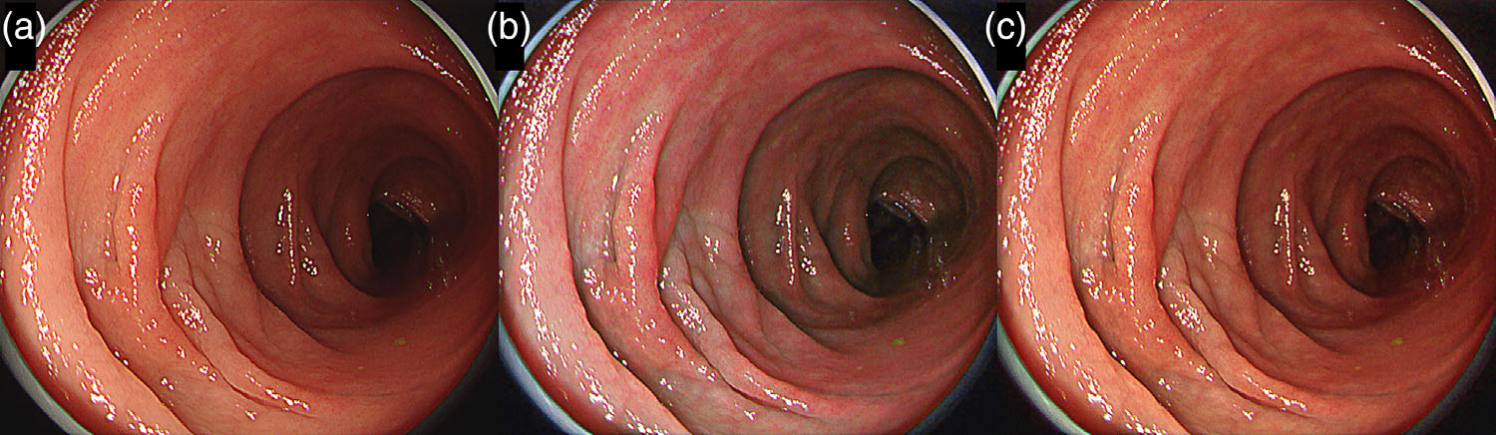
Intramucosal adenocarcinoma (IIa) lesion located in the transverse colon. (a) WLI; (b) TXI mode1; (c) TXI mode2. WLI: White light imaging; TXI: texture and color enhancement imaging

Figure 3 and Video 1 show an intramucosal adenocarcinoma (IIa) lesion located in the transverse colon visualized using WLI, TXI mode1, and TXI mode2.

### Statistical analysis

Statistical analysis was performed using Minitab for Windows (Minitab 19 2019; Pennsylvania, USA). Normally distributed parametric data are expressed as mean (± SD), and non‐parametric data are expressed as the median (range). To determine differences in the visualization scores, comparisons among the groups were performed by one‐way analysis of variance, followed by multiple comparison testing using the Bonferroni–Dunn method. Normally distributed parametric data were analyzed using a *t*‐test. A *p*‐value <0.05 was considered statistically significant and a *p*‐value <0.1 was interpreted as a tendency.

## RESULTS

Overall, 22 patients were recruited and enrolled in this study. In total, 68 lesions were detected during colonoscopy, and all lesions were resected endoscopically. The median patient age was 63 (37–87) years, the male‐to‐female ratio was 1:0.47, and the mean lesion size was 7.1 (±4.79) mm. Histologically, of the 68 lesions, there were eight hyperplastic polyps (11.8%), six sessile serrated lesions (8.8%), 46 tubular adenomas (67.6%), six mucosal cancers (8.8%), and 2 submucosal tumors (2.9%). Regarding macroscopic types, 41 lesions were flat elevated (60.3%) and 27 were protruded/others (39.7%). Tumor location showed 38 lesions (55.9%) in the right colon (proximal to the splenic flexure) and 40 (44.1%) in the left colon (distal to the splenic flexure; including rectum) (Table 1). Finally, video files for evaluation consisting of 272 randomly arranged video clips were produced.

**TABLE 1:** Patient and lesion characteristics

Number of patients	22
Number of lesions	68
Age, median (range)	63 (37–87)
Sex male/female	15/7
Size of the lesion, Mean (±SD)	7.16 mm (±4.79)
Histology of the lesion (hyperplastic/SSL/tubular adenoma/mucosal adenocarcinoma/SMT)	8/6/46/6/2
Macroscopic type of the lesion (IIa/IIc/Is/Ip/others)	40/1/22/3/2
Location of the lesions (Right; proximal to the splenic flexure /Left; distal to the splenic flexure, including rectum)	38/30

Abbreviations: SMT, Submucosal tumor; SSL, Sessile serrated lesion.

**FIGURE 4:**
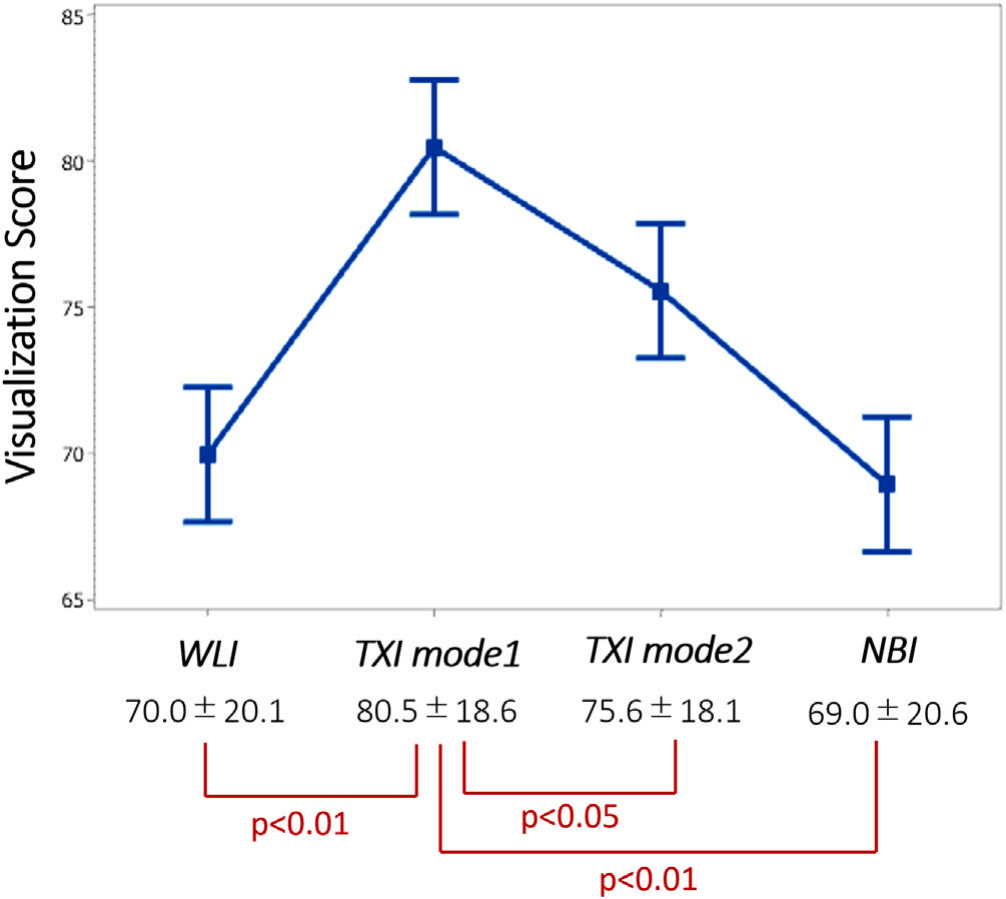
Visualization scores among WLI, texture and TXImode1, TXImode2, and NBI. WLI: White light imaging; TXI: texture and color enhancement imaging; NBI: narrow‐band imaging

**FIGURE 5:**
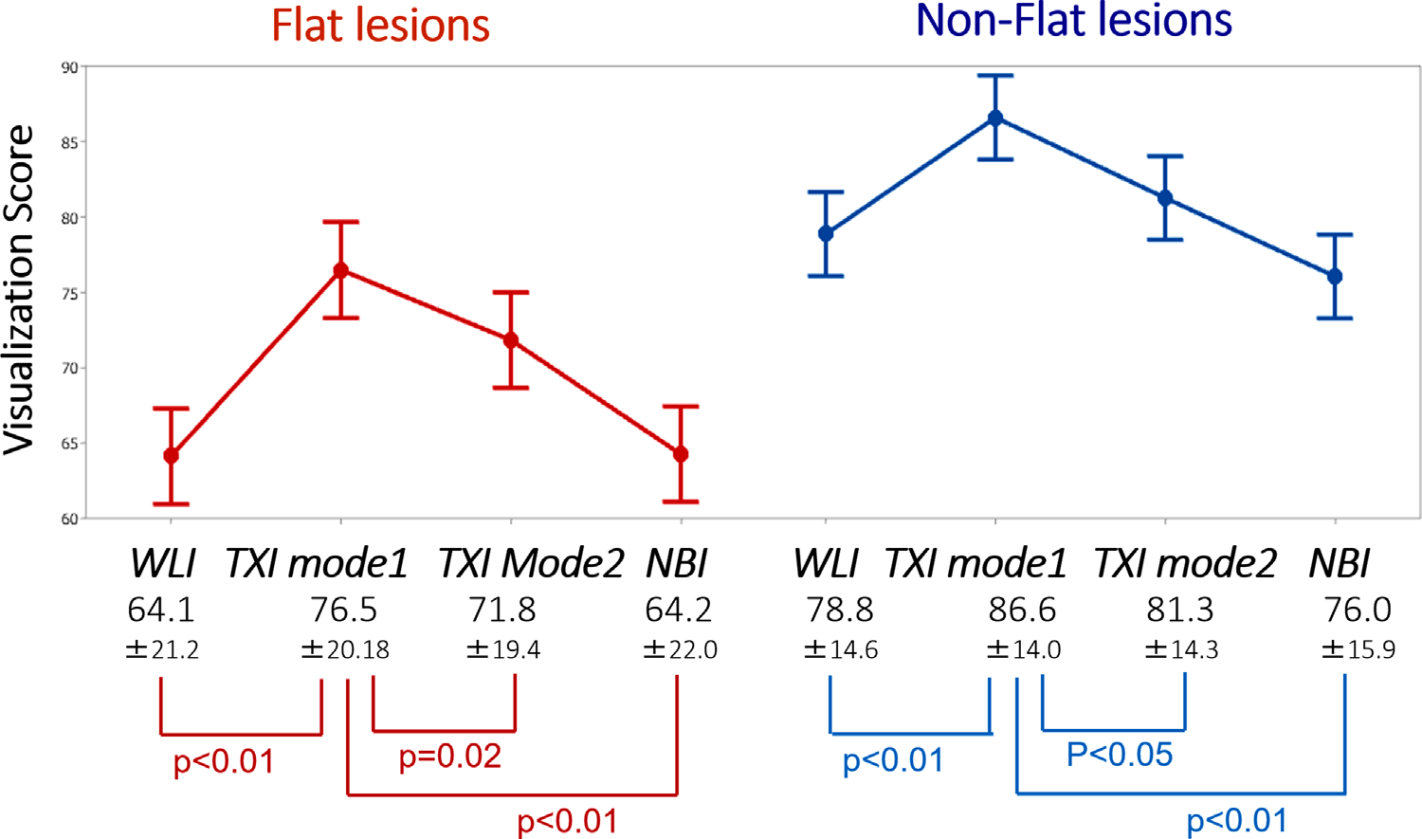
Visualization scores among WLI, TXImode1, TXI imaging mode2, and NBI of flat and non‐flat lesions. WLI: White light imaging; TXI: texture and color enhancement imaging; NBI: narrow‐band imaging

The mean visualization scores among WLI, TXI mode1, TXI mode2, and NBI were 70.0 (±20.1), 80.5 (±18.6), 75.6 (±18.1), and 69.0 (±20.6) (Figure 4), respectively. The visualization score of TXI mode1 was significantly higher than that of WLI, TXI mode2, and NBI. Regarding the flat lesions, mean visualization scores among WLI, TXI mode1, TXI mode2, and NBI were 64.1 (±21.2), 76.5 (±20.18), 71.8 (±19.4), and 64.2 (±22.0) (Figure 5), respectively. The visualization score of TXI mode1 was significantly higher than that of WLI, TXI mode2, and NBI in flat lesions. Regarding the serrated lesions, WLI, TXI mode1, TXI mode2, and NBI were 65.9 (±22.0), 72.7 (±23.0), 66.6 (±22.1), and 58.2 (±25.0), respectively.

## DISCUSSION

This report is the first to evaluate the efficacy of TXI for improving the visibility of colorectal lesions, although visibility evaluation of early gastric cancer, pharynx, and esophageal carcinoma suspicious lesions have been reported.^16^, ^17^ The advancement of IEE technology allows us to differentiate colorectal lesions and estimate the invasion depth of colorectal cancers^18^, ^19^, ^20^, ^21^; however, whether IEE can detect more colorectal lesions or increase adenoma detection rate (ADR) is still controversial,^8^, ^9^, ^10^, ^11^, ^12^, ^13^ although one meta‐analysis revealed LCI can improve the ADR^22^ One of the IEE techniques that could potentially enable an increase in ADR is NBI. Recently, meta‐analysis clarified that NBI can increase the ADR of patients with the best bowel preparation; however, the best preparation rate of this meta‐analysis was limited to 20%.^23^ Therefore, the development of a technique that can increase the ADR of all patients is still required. To reduce the rate of missed lesions in the visual field, brighter and adequate light‐distributed images, with enhanced color, and the ability to discern differences in surface structure are required. TXI, a novel IEE, enables the brightening of dark areas of WLI, enhances the texture analysis, including those with subtle surface elevation or depression, and enhances color differences between the surrounding mucosa of colorectal lesions. Therefore, TXI seems to be a promising technology for improving the detectability of colorectal lesions in the visual field.

This study clarified that TXI enables improved visualization of colorectal lesions, both protruded and flat. TXI, especially TXI mode1, allows better visualization of colorectal neoplasia than WLI and NBI, and this result implies that TXI can detect more colorectal lesions.

Small sample size, potential selection bias, single‐center study, subjective evaluation method, use of reconstructed video, non‐blinded study, omission of sample size calculation, and video clips of NBI recorded from non‐identical viewpoint should be considered as the limitation of this study. In addition, improvement of the visibility of colorectal lesions might not allow better detection of colorectal lesions.

In conclusion, our study demonstrated that the novel IEE TXI enables improved visualization of colorectal lesions compared to WLI and NBI for both protruded and flat lesions. TXI may allow better detection of colorectal lesions, although further prospective study is required.

## CONFLICT OF INTEREST

Kazuki Sumiyama is a Deputy Editor‐in‐Chief of DEN Open. The rest of the authors do not have any conflict of interest.

## FUNDING INFORMATION

This study received no specific grant from any funding agency in the public, commercial, or not‐for‐profit sectors.

## Supporting information


**Video1**. Constructed videos of Intramucosal adenocarcinoma (IIa) lesion located in the transverse colon visualized using WLI, TXI mode1, and TXI mode2.
